# Supplementary material for: Disulfiram Inhibits Opsonin-Independent Phagocytosis and Migration of Human Long-Lived In Vitro Cultured Phagocytes from Multiple Inflammatory Diseases
Source: Cells. 2024 Mar 18;13(6):535. doi: 10.3390/cells13060535 (PMC10968875; doi:10.3390/cells13060535)
Supplement: Supplementary file 1 [file cells-13-00535-s001.zip › cells-2861608-supplementary.pdf]

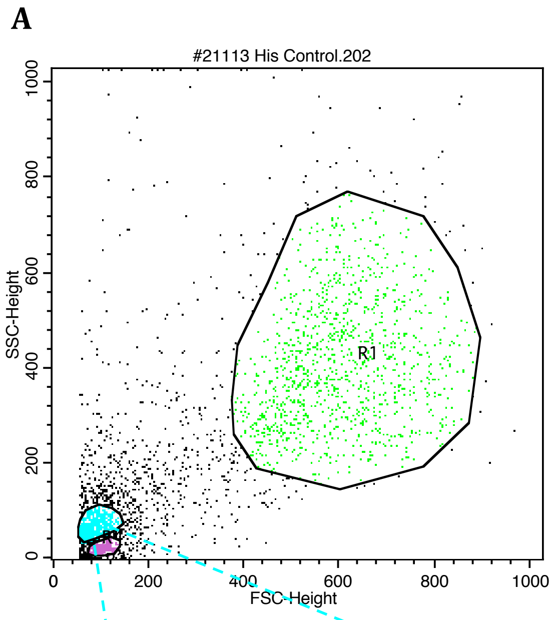

B

	Gated %	CD45 %	CD63 %
Exosomes (blue)	20.76	3.19	50.97

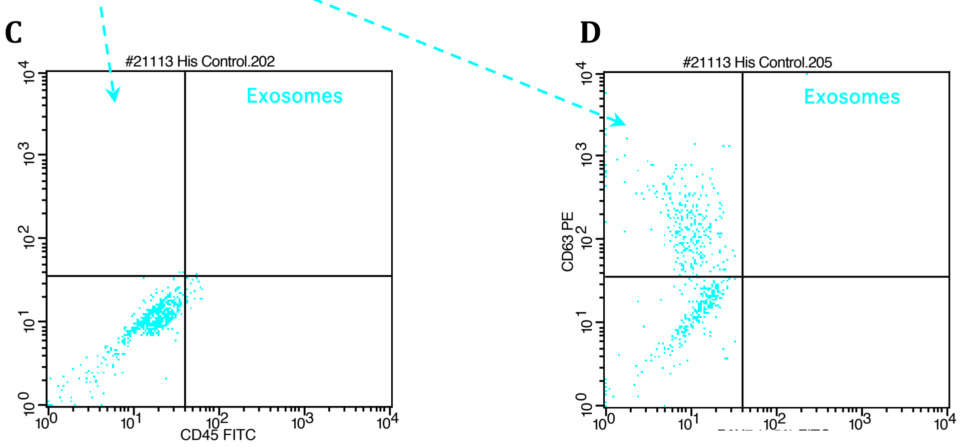

Supplementary Figure S1. Gating strategy for the exosomes population in cultured phagocytes. Human phagocytes were cultured for 3-4 weeks and were stained by leukocyte common antigen CD45 and intracellular vesicle marker CD63 measured by flow cytometry. Dots plot (**A**) shows populations in cell culture containing macrophages (green), lymphocytes (purple), and exosomes excreted by macrophages (blue). Percentage of quantity, CD45, and CD63 of exosomes was presented in table **B**. Exosomes were characterized by the negative expression of CD45 (**C**, x-axis) and major population with positive expression of CD63 (**D**, y-axis).

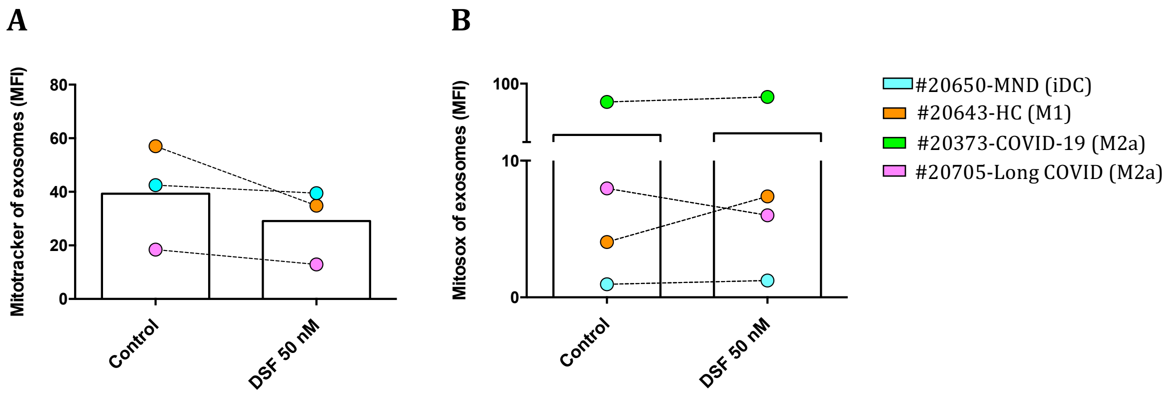

Supplementary Figure S2. Effect of DSF on mitochondrial superoxide, mitochondria mass in exosomes potentially secreted by general macrophages and dendritic cells. Phagocytes were cultured for 3-4 weeks and treated with DSF 50 nmol.L-1 or DMSO as vehicle control (0.01% v/v final concentration) for 24 h at 37°C and 5% CO2. MFI of Mitotracker (A), Mitosox (B) of exsosomes secreted by macrophages and dendritic cells were examined using flow cytometry. Data are visualized as mean from n≥ 3 cell cultures. Diagnoses of donors classified by macrophages and

dendritic cells are labeled with colored symbols. HC: healthy control; MND: motor neuron disease; COVID-19: coronavirus disease; MFI: mean fluorescence intensity.

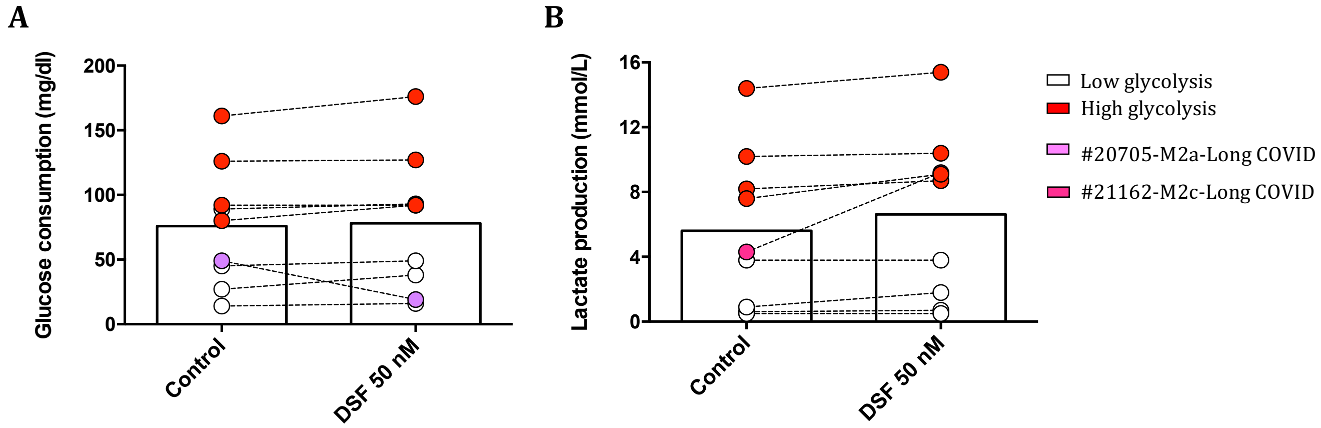

Supplementary Figure S3. Effect of DSF on glucose and lactate metabolism of long-lived phagocyte cultures. Glucose consumption (y-axis determined by subtracting glucose determined in the respective phagocyte culture from the glucose contents of the culture medium) is shown for 9 phagocyte cultures in (**A**). Lactate production (y-axis determined by subtracting the lactate contents of the culture medium from lactate determined in the respective phagocyte culture) of the respective phagocyte cultures are shown in (**B**). DSF decreased glucose consumption in #20705 in one cell culture which was already low in glucose consumption in (**A**) while increased lactate production of #21162 in (**B**). Both of them were derived from different Long-COVID patients. Residual cultures did not show a remarkable effect by DSF treatment. Open circles signify phagocytes with low lactate metabolism; red-filled circles signify phagocytes with high lactate metabolism.

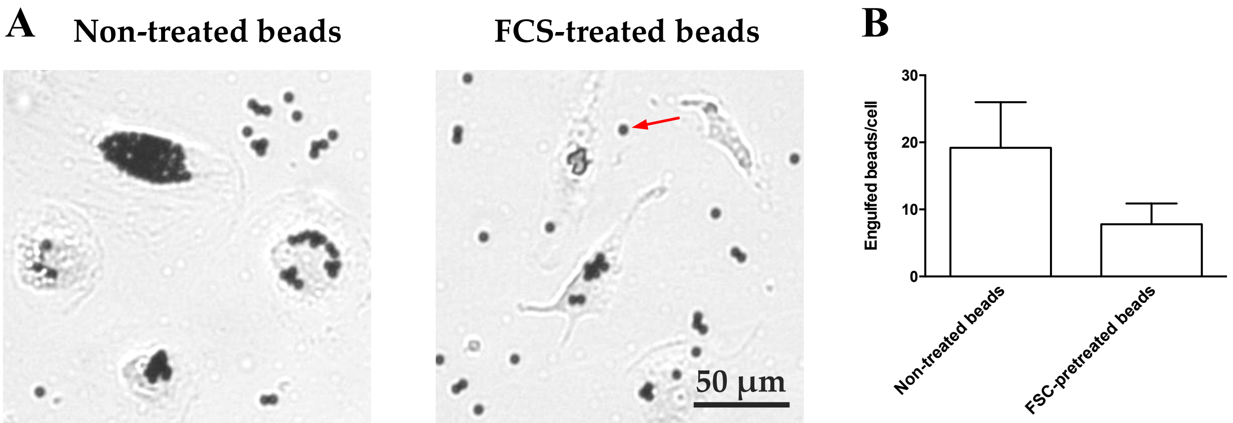

Supplementary Figure S4. Phagocytic capacities of representative macrophages for non-opsonized Dynabeads® M-450 Epoxy with and without FCS pre-treatment. Phagocytes derived from patients #21035-MDD, #21074-CFS, and #21079-CFS were cultured for 3 weeks and incubated with Dynabeads® M-450 Epoxy with and without FCS pretreatment at a ratio of 20 beads per cell for a 24-hour video recorded by Juli™ Br at 37°C. **A.** Images of non-opsonized Dynabeads® M-450 Epoxy with and without FCS pretreatment engulfed by phagocytes (#21035). Images were phase-contrast images at 4× objective obtained by Juli™ Br after 24 h incubation of macrophages. The red arrow indicates a single bead. Scale bar: 50 µm. **B.** Phagocytic capacities for non-opsonized Dynabeads® M-450 Epoxy with and without FCS pretreatment by phagocytes in 24 h. Data are visualized as mean ± SEM from n=3 phagocyte cultures for the amount of ingested beads per cell. The number of engulfed beads by phagocytes was counted manually. MDD: major depressive disorder, CFS: chronic fatigue syndrome, FCS: fetal calf serum.
